# Fatal myocarditis in a child with systemic onset juvenile idiopathic arthritis during treatment with an interleukin 1 receptor antagonist

**DOI:** 10.1186/1546-0096-10-8

**Published:** 2012-04-10

**Authors:** Andrew S Zeft, Shaji C Menon, Dylan Miller

**Affiliations:** 1 Children's Hospital, Cleveland Clinic, Pediatric Rheumatology, 9500 Euclid Avenue/A111, Cleveland, OH 44195, USA; 2 Division of Pediatric Cardiology, Department of Pediatrics, University of Utah, Salt Lake City, UT 84158, USA; 3 Department of Pathology, Intermountain Medical Center, Salt Lake City, UT 84158, USA

**Keywords:** Arthritis, Myocarditis, Idiopathic, Interleukin, Juvenile

## Abstract

**Background:**

The pathologic diagnosis of isolated myocarditis without pericardial involvement is uncommonly encountered in systemic onset Juvenile Idiopathic Arthritis (soJIA).

**Case:**

An eleven year-old boy with soJIA died suddenly while being treated with the interleukin 1 (IL-1) receptor inhibitor, anakinra. His autopsy revealed an enlarged heart and microscopic findings were consistent with myocarditis, but not pericarditis. Viral PCR testing performed on his myocardial tissue was negative.

**Conclusion:**

This case illustrates myocarditis as a fatal complication of soJIA, potentially enabled by anakinra.

## Background

Systemic onset juvenile idiopathic arthritis (soJIA) is a chronic auto-inflammatory disease of childhood characterized by quotidian fever, evanescent rash, serositis, lymphadenopathy, splenomegaly, and synovial joint inflammation [1]. Isolated myocarditis without pericardial involvement has been described in SoJIA [2], and individuals with active soJIA have been described with signs of heart failure in the absence of overt pericardial effusion [3]. Cardiac death has occurred in a patient with adult-onset Still's disease treated with IL-1 receptor inhibitor, anakinra [4]. We present a child with soJIA who died unexpectedly while receiving anakinra whose autopsy revealed an inflammatory myocarditis without pericarditis.

## Case presentation

A 10 year-old adopted, African American boy with a history of autism and asthma presented to our pediatric rheumatology clinic with a pruritic evanescent erythematous macular rash, polyarticular large and small joint arthritis, subjective fever in a quotidian pattern, 10 pound weight loss and fatigue, and cervical lymphadenopathy. His electrocardiogram was within normal limits. His blood work revealed leukocytosis with a predominance of neutrophils (WBC 17 K/μL, neutrophils 84%), normocytic anemia (Hbg 9.9 g/dL), mild thrombocytosis (452 K/μL), and normal creatinine, uric acid, LDH, and complement levels. Signs of systemic inflammation were evident (ESR 80 mm/h, CRP 14 mg/dL, d-dimer 2890 ng/mL, soluble IL-2 receptor 1058 U/mL, ferritin 673 ng/mL). His urine revealed 12 RBC's, mild microscopic hematuria, and normal calcium creatinine ratio. An abdominal CT scan was recommended but not performed. Autoantibody testing was negative (RF, CCP, ANA, dsDNA, Smith, ANCA, antiGBM) and his HLA-B27 allele was negative. A diagnosis of soJIA was made based on International League Against Rheumatism criteria [5].

As is common in patients with soJIA, he had a waxing and waning disease course [1]. Five months following presentation, on NSAID, prednisone 0.17 mg/kg/day, and anakinra 1.2 mg/kg/day, he developed acute, positional chest and shoulder pain, clinically consistent with pericarditis. An oral decadron pulse (60 mg/m^2^) divided over 3 days induced quick resolution of his symptoms. His anakinra dose was increased to 1.6 mg/kg/day. An echocardiogram was recommended but not performed. Arthritis, intermittent periorbital edema, and laboratory evidence of active disease persisted and seven months following presentation methotrexate was added which was not tolerated and discontinued. Nine months following presentation bilateral ankle and knee intra-articular corticosteroid injections (total aristospan 94 mg) were performed, and he was started on cyclosporine. Of note, he was asymptomatic with a normal laboratory evaluation 15 months into his disease course. Follow up urine analysis was clear suggesting that chronic nonsteroidal anti-inflammatory exposure may have precipitated his hematuria [6].

However, 6 weeks later, on NSAID, prednisone 0.33 mg/kg/day, low dose cyclosporine 0.36 mg/kg/day, and anakinra 1.4 mg/kg/day his arthralgias, morning stiffness, and fatigue returned. He described having morning emesis (up to 3 per week), upper respiratory congestion, sore throat, and self-resolving headaches. Two weeks later his morning stiffness worsened, so his anakinra dose was divided into morning and evening doses (0.7 mg/kg/dose). One week later he represented to an urgent care clinic complaining of cough, evening chest pain, wheeze, upper respiratory congestion, poor appetite, and mild nausea. He had low-grade fever (38.2°C), mild tachycardia (124 beats/min), an otherwise normal physical examination, and his chest radiograph revealed left lower lobe interstitial markings and peribronchial cuffing. He was assessed to have a viral illness. Two days following he returned, now with emesis and diarrhea. His temperature was 37.7°C, and he had mild tachycardia (114), tachypnea (24 breaths/min), and hypertension (132/83). He had scattered wheezes, without other abnormalities on physical exam. Report of his chest x-ray documented his heart size within "the upper limits of normal". At home two days later, he was found apneic and pulseless. Unsuccessful attempts at resuscitation were made, during which an urgent bedside echocardiogram did not identify pericardial effusion. An autopsy was performed.

## Pathology

**Figure 1:**
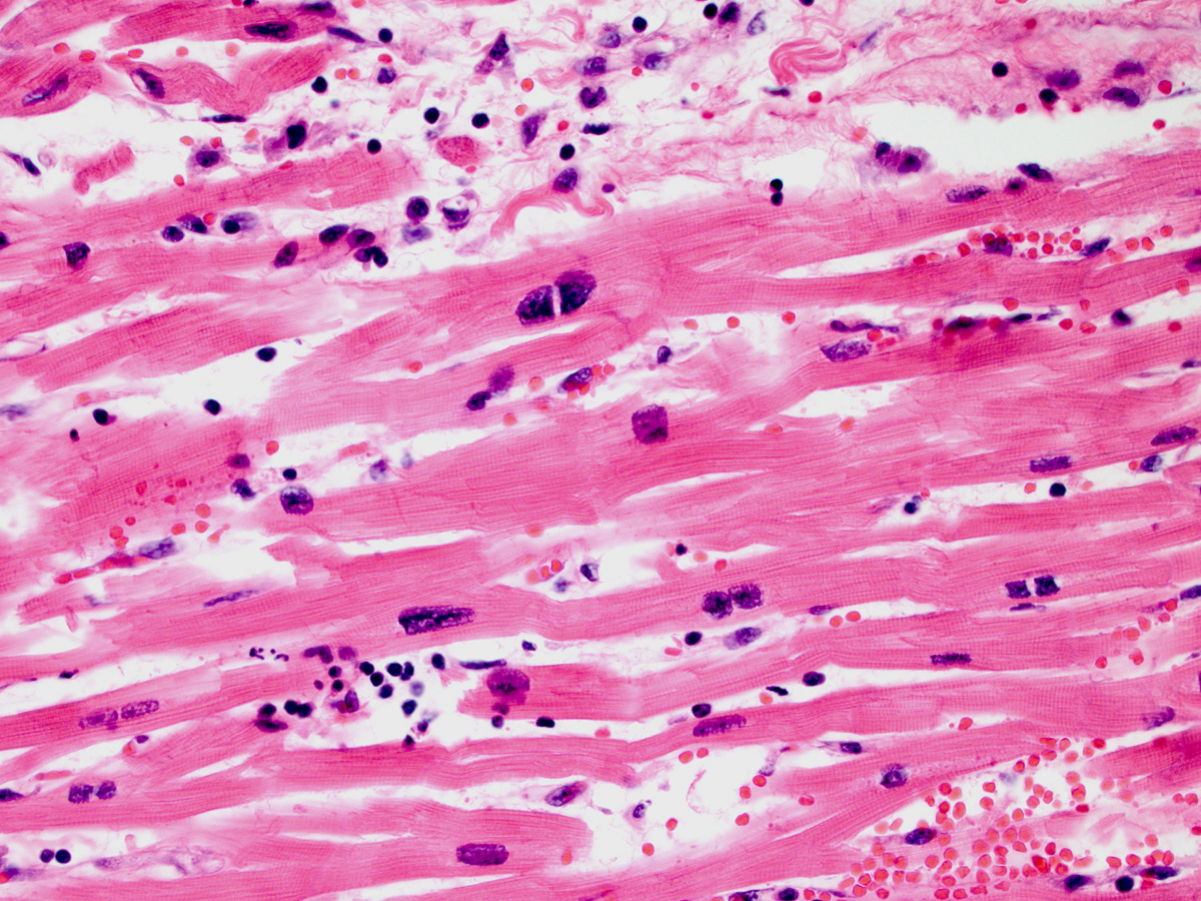
**Myocarditis with predominantly lymphocytic inflammation**. Photomicrograph (H&E, ×400) showing interstitial inflammatory cells consisting mostly of small lymphocytes with round nuclei and scanty cytoplasm, though rare granulocytes are also seen. This image also shows several binucleated myocytes, a feature of myocyte hypertrophy, though this was not a consistent finding elsewhere.

**Figure 2:**
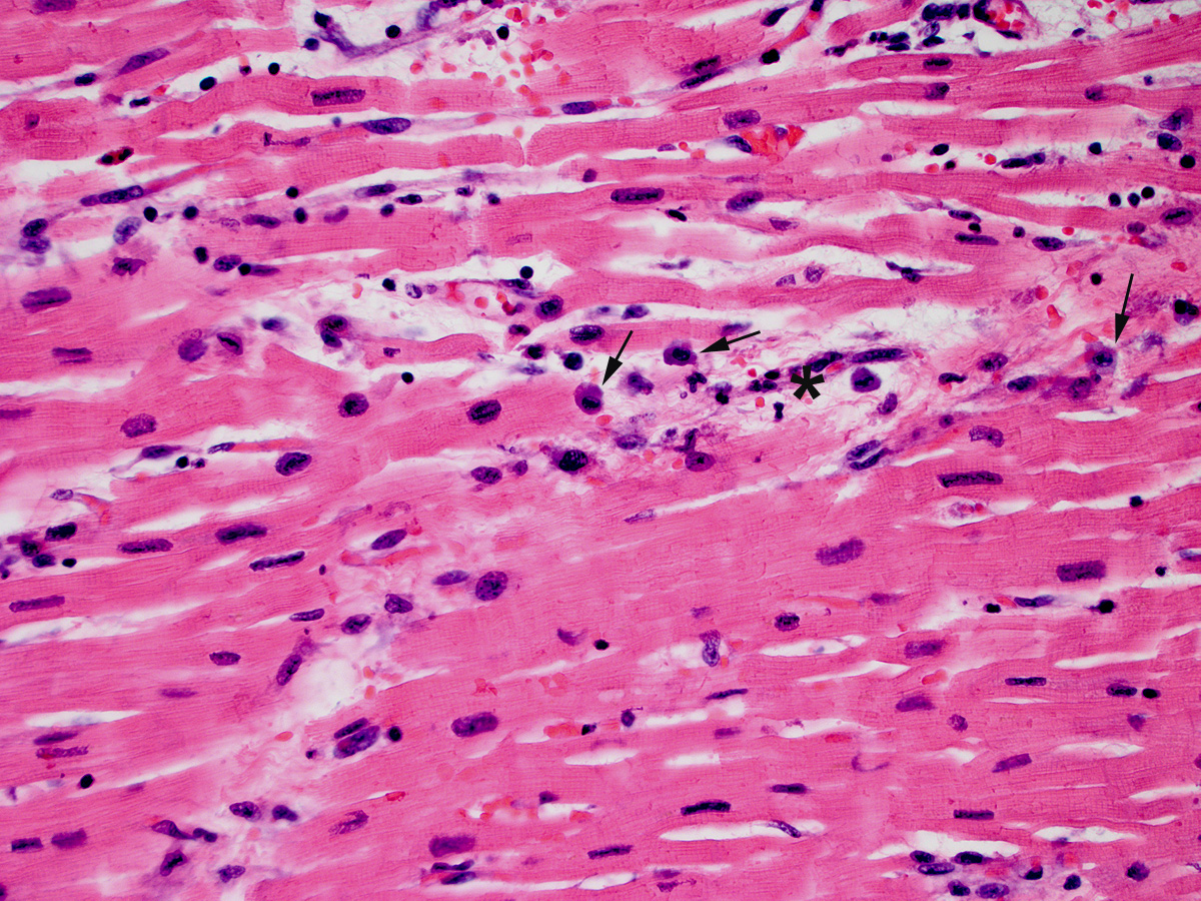
**Myocarditis with focal myocyte injury and macrophage infiltration**. Photomicrograph (H&E, ×400) showing a focus of myocyte injury. A necrotic myocyte is highlighted (asterisk) with surrounding macrophages (arrows) having more abundant cytoplasm.

**Figure 3:**
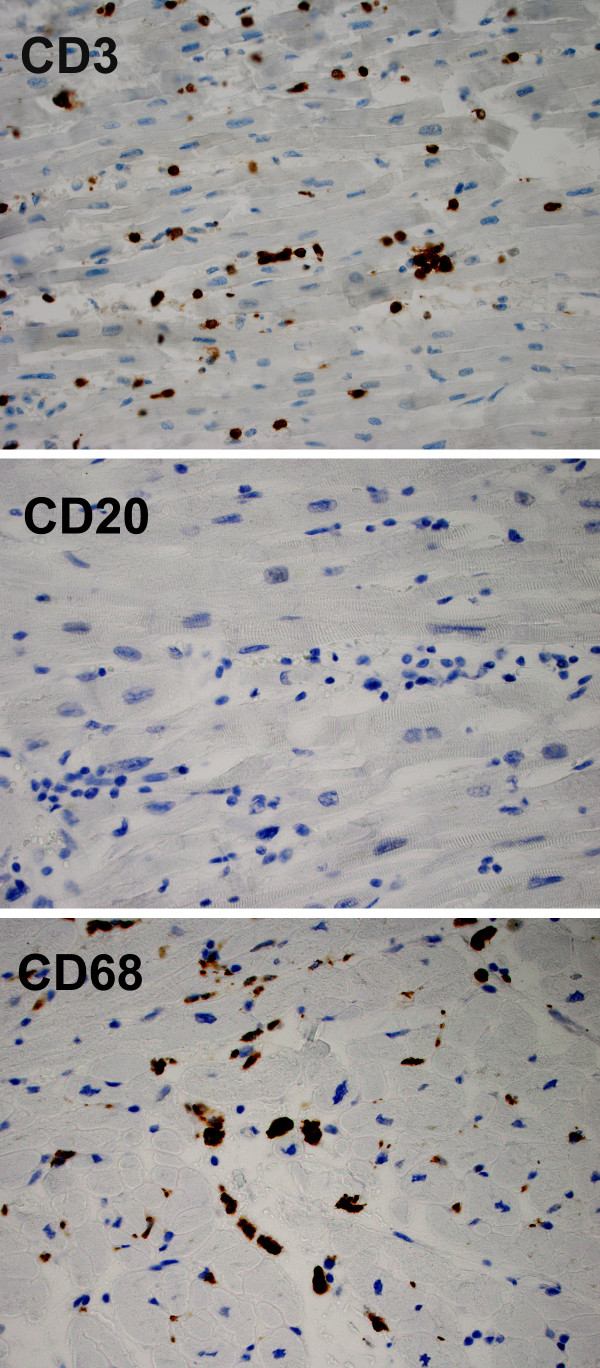
**Immunophenotyping of inflammatory cells in the myocardium**. Photomicrographs (×400) showing immunohistochemical staining of paraffin-embedded formalin-fixed myocardial tissue sections using antibodies directed against CD3, CD20, and CD68. The inflammatory cells are mostly CD3 positive T-cells and CD68 positive macrophages. CD20 positive B-cells were essentially absent.

At autopsy, the heart weighed 440 grams (expected: 250-430 grams), with smooth, glistening pericardial surfaces without adhesions (no pericarditis) and no excess pericardial fluid. The myocardium was grossly unremarkable, but showed edema microscopically with extensive infiltration by a mixed inflammatory cell infiltrate including predominantly T-lymphocytes [Figures 1, 2 and 3] as well as areas of myocyte damage with infiltrating macrophages [Figures 2 and 3]. There was no histologic evidence of pericarditis. Examination of the coronary arteries revealed no evidence of vasculitis. Electron microscopy of the myocardium demonstrated only nonspecific degenerative changes with intact sarcolemmal membranes throughout. The enlargement of the heart likely reflected edema as histologic features of cardiomyopathy (such as fibrosis, and myocyte hypertrophy and disarray) were absent. No significant changes were seen in the lungs, spleen, and liver. The kidneys showed evidence of mild acute tubular necrosis.

Myocardial tissue was PCR negative for potential viral etiologies (Adenovirus, Cytomegalovirus, Epstein-Barr Virus, Enteroviruses including Coxsackie, Parvovirus).

## Discussion

More than three decades ago, the prevalence of death in juvenile rheumatoid arthritis (JRA) was examined and fatal cases occurred most frequently in patients with systemic JRA [7,8]. Mortality associated with soJIA is typically caused by Macrophage Activation Syndrome or infectious complications related to immune suppressive therapy. The association of myocarditis with soJIA and adult-onset Still's disease has been described, but its prevalence in this disease is unclear since myocarditis may be subclinical and escape recognition by electrocardiography and echocardiography [3,9-16]. Furthermore, the definitive diagnosis of myocarditis requires microscopic evaluation of myocardial tissue. In soJIA, pericarditis typically accompanies myocarditis, but isolated myocarditis (as seen in this patient) has also been described [3].

Our patient had symptoms of viral illness, and autopsy revealed patchy peribronchial inflammation. Thus, it is possible that a virus or other infectious agents, such as parainfluenza, Human herpes virus 6, or Mycoplasma, not tested for by our PCR methods precipitated the inflammatory myocardial changes found on autopsy [17,18]. Unfortunately, the morphologic appearance of myocarditis is frequently identical for viral, post-viral, bacterial, and drug-related etiologies. The features observed in our patient were most consistent with lymphocytic myocarditis with predominantly T-cells and associated myocyte injury. While macrophage activation syndrome has occurred in soJIA patients on anakinra [19], our patient's autopsy did not identify signs of severe systemic involvement outside of inflammatory myocardial disease.

Numerous case series have described anakinra's efficacy in treating the systemic features of soJIA [19-21], although some patients have persistent disease while being treated with anakinra. Immune suppression from anakinra, prednisone, and cyclosporin could have facilitated infection induced myocardial inflammation in this patient. Alternatively, incompletely controlled and clinically undetected myocarditis from soJIA could have facilitated an infection-induced inflammatory response in the myocardium. To our knowledge there have been no soJIA patients on anakinra who have been described with biopsy proven myocarditis, and the package insert for anakinra does not list cardiac related adverse events. Cardiac death has been reported, however, in a patient with adult-onset Still's disease treated with IL-1 receptor inhibitor anakinra [4]. Her autopsy revealed a dilated cardiomyopathy as the most probable cause of death, while histologic evaluation showed no signs of ischemia, myocardial infarction, or viral myocarditis.

The systematic collection of data to assess possible associations between IL-1 blockade and cardiac complications is very important, especially since anakinra and longer acting IL-1 inhibitors are now given more frequently in auto-inflammatory conditions [22,23]. Currently, a multi-centered North American study is examining the rate of severe adverse events in JIA, which will allow for better understanding of the spectrum of disease and potential medication related side effects [24].

## Conclusion

This case illustrates myocarditis as a fatal complication of soJIA, potentially enabled by anakinra.

## Consent

Consent for publication has been obtained from the parents of the patient.

## Competing interests

The authors declare that they have no competing interests.

## Authors' contributions

AZ is the primary author of the manuscript. SM made substantial contributions to the interpretation of data, revised the manuscript critically, and gave final approval of the version to be published. DM made substantial contributions to acquisition and interpretation of data, revised the manuscript critically, and gave final approval of the version to be published.
